# Non-typhoidal Salmonella Bacteremia as a Diagnostic Clue to Gastric Cancer With Isolated Bone Metastasis: A Case Report

**DOI:** 10.7759/cureus.87147

**Published:** 2025-07-01

**Authors:** Ana Rita Antunes, Mariana Teixeira, Rita Menezes Azevedo, Inês Amaral Pinto, Andreia Freitas

**Affiliations:** 1 Internal Medicine, Unidade Local De Saúde Gaia E Espinho, Vila Nova De Gaia, PRT; 2 Critical Care Medicine, Unidade Local De Saúde Gaia E Espinho, Vila Nova De Gaia, PRT

**Keywords:** gastric adenocarcinoma, immunosuppression, isolated bone metastasis, lumbar pain, non-typhoidal salmonella bacteremia

## Abstract

We report a rare case of *Salmonella enterica* bacteremia as the initial presentation of gastric adenocarcinoma with isolated bone metastasis. A 65-year-old woman presented with persistent lumbar and cervical pain, night sweats, tremors, and worsening respiratory failure. Blood cultures grew *S. enterica*, leading to targeted antibiotic treatment with ceftriaxone. Despite therapy, fever and inflammatory markers persisted. Upper endoscopy revealed an ulcerated gastric lesion, and biopsy confirmed signet ring cell gastric adenocarcinoma. Imaging showed diffuse vertebral lesions, and bone biopsy confirmed metastatic disease. The patient’s condition was complicated by gastrointestinal bleeding and progressive decline, making curative treatment unfeasible; she was transitioned to palliative care. This case highlights non-typhoidal *Salmonella* (NTS) bacteremia as a possible early indicator of malignancy and emphasizes the need for thorough diagnostic evaluation in patients with unexplained bacteremia.

## Introduction

*Salmonella* is a gram-negative bacterium belonging to the Enterobacteriaceae family, consisting of two species: *Salmonella bongori* and *Salmonella enterica* [1]. Non-typhoidal *Salmonella* (NTS) primarily causes self-limited gastroenteritis; however, invasive disease with bacteremia occurs in approximately 3%-8% of cases, especially in immunocompromised individuals [2-4]. Risk factors for invasive NTS infection include connective tissue diseases, therapeutic immunosuppression, malignancies, diabetes mellitus, and human immunodeficiency virus (HIV) infection [2-5]. Among these, malignancy and HIV infection are the most frequent underlying conditions in patients with NTS bacteremia and are associated with higher in-hospital mortality [4,5].

Patients with hematologic malignancies have a five to ten-fold increased risk of *Salmonella* infection compared to those with solid tumors, where the risk increases by only 1-1.5 times [6]. Among patients with solid tumors, gastrointestinal malignancies are among the most frequently associated with NTS bacteremia [7]. Given that primary bacteremia without gastroenteritis is more commonly observed in severely immunosuppressed patients, a thorough evaluation for underlying immunosuppressive conditions is warranted in such cases.

Gastric cancer ranks as the fifth most frequently diagnosed malignancy and the fourth leading cause of cancer-related mortality worldwide, accounting for 8.2% of all cancer-associated deaths [8,9]. Its clinical presentation varies depending on tumor location, size, and disease stage [10-12]. Early-stage gastric cancer is often asymptomatic or presents with nonspecific symptoms, while advanced disease commonly manifests as weight loss, dyspepsia, early satiety, dysphagia, and anemia due to chronic occult bleeding [8-12]. Metastatic involvement may present with lymphadenopathy or jaundice [8-12].

Despite advances in medical care, nearly 50% of patients are diagnosed after the disease has already spread beyond the locoregional area [12]. Gastric cancer metastasizes through direct invasion, lymphatic dissemination, hematogenous spread, or peritoneal seeding. The liver, peritoneum, and lymph nodes are the most common metastatic sites, whereas less frequent sites include the lungs, bones, ovaries, and, in rare cases, the colon [10-12].

We report an exceptionally rare case of NTS bacteremia serving as a diagnostic clue to a rare presentation of gastric cancer with isolated bone metastasis. This case highlights the importance of a comprehensive investigation in patients with NTS bacteremia, as it may reveal an underlying malignancy.

## Case presentation

A 65-year-old woman with a medical history of hypertension, hypercholesterolemia, and obesity presented to the emergency department with persistent and uncontrolled lumbar and cervical pain lasting several weeks. She also reported night sweats and tremors for one week but denied fever. One month earlier, she had been evaluated by her primary care physician for similar symptoms and prescribed ibuprofen 400 mg. Due to ongoing pain, she sought emergency care one week later but was discharged with tramadol, dexketoprofen, and metoclopramide. The patient denied any trauma to the lumbar or cervical spine, neurological deficits, additional pain sites, weight loss, gastrointestinal or urinary symptoms, dyspnea, recent travel, or animal contact. Social and family histories were unremarkable, and she lived independently.

On examination in the emergency department, she was alert and cooperative but exhibited markedly dry mucous membranes. Vital signs revealed blood pressure of 166/94 mmHg, heart rate of 114 beats per minute with a regular rhythm, auricular temperature of 37.7°C, and respiratory rate of 24 breaths per minute with accessory muscle use. Peripheral oxygen saturation was 90% while receiving oxygen via nasal cannula at two liters per minute. Lung auscultation revealed globally diminished breath sounds bilaterally, with no adventitious sounds, and cardiac auscultation was unremarkable. The abdomen was distended but soft, with mild tenderness in the left quadrants. No peripheral edema or skin lesions were noted.

Initial investigations revealed type I respiratory insufficiency with a partial oxygen pressure (PaO₂) of 48.9 mmHg (normal range 75-100 mmHg), partial carbon dioxide pressure (PaCO₂) of 28.5 mmHg (normal range 35-45 mmHg), oxygen saturation of 88% (normal range 95%-100%), and a PaO₂/fraction of inspired oxygen ratio of 232.9 on room air. Blood tests showed microcytic, hypochromic anemia, thrombocytopenia, elevated alkaline phosphatase, and raised C-reactive protein (Table 1). Urinalysis and urinary antigen testing for *Legionella pneumophila* and *Streptococcus pneumoniae*, along with nasopharyngeal antigen testing for SARS-CoV-2, influenza A and B, and respiratory syncytial virus, were all negative. Abdominal ultrasound and computed tomography (CT) showed no abnormalities, while chest CT angiography excluded pulmonary thromboembolism.

**Table 1:** Laboratory test results at the time of admission to the emergency department

Parameters	Patient values	Reference range
Hemoglobin	10.9	12.0-16.0 g/dL
Mean corpuscular volume	82.6	80.0-100.0 fL
Mean corpuscular haemoglobin concentration	33.7	32.0-36.0 g/dL
White blood cells	5020	3600-11.000 uL
Platelets	21	150-440 uL
Immature platelet fraction	7.2	1.1%-7.1%
Urea	82	17.0-50.0 mg/dL
Creatinine	0.55	0.51-0.95 mg/dL
Sodium	138	136.0-145.0 mmol/L
Potassium	4.07	3.5-5.0 mmol/L
Chloride	105.2	98.0-107.0 mmol/L
Aspartate aminotransferase	55	4.0-27.0 U/L
Alanine aminotransferase	38	4.0-34.0 U/L
Alkaline phosphatase	555	35.0-104.0 U/L
Pancreatic amylase	51	13.0-53.0 U/L
Total Bilirrubin	0.88	0.1-1.1 mg/dL
Lipase	38	13.0-60.0 U/L
Troponin	10	5.0-14.0 ng/L
C-reactive protein	28.88	0-0.5 mg/dL

Due to worsening respiratory failure requiring increased oxygen supplementation, neurological deterioration with prostration, and severe thrombocytopenia, she was admitted to the intermediate care unit for management of sepsis of unknown origin. Empirical ceftriaxone was initiated. During seven days in intermediate care, she received supportive treatment for multiorgan dysfunction. Initially on high-flow nasal cannula, she was escalated to continuous positive airway pressure, resulting in improved respiratory function and subsequent weaning to nasal cannula. Blood cultures yielded *S. enterica* susceptible to cefotaxime and ceftriaxone, and targeted therapy with ceftriaxone was continued.

With organ function improvement, she was transferred to the internal medicine ward for ongoing care and continuation of the etiological workup. The identification of *S. enterica* bacteremia in a patient without a history of known immunosuppression prompted a comprehensive etiological investigation that included an assessment for potential extraintestinal foci of infection. Extensive infectious disease screening, including tests for HIV, *Listeria monocytogenes, Leptospira spp., Rickettsia conorii, Coxiella burnetii, Borrelia spp*., and *Brucella* (Rosa Bengala test), yielded negative results. Cytomegalovirus serology was consistent with past infection. Protein electrophoresis revealed polyclonal hyperglobulinemia. Serum calcium was within normal limits, and complement levels showed no evidence of consumption. Immunoglobulin levels, light chains, and serum immunofixation were within normal ranges. Echocardiography ruled out endocarditis.

**Figure 1:**
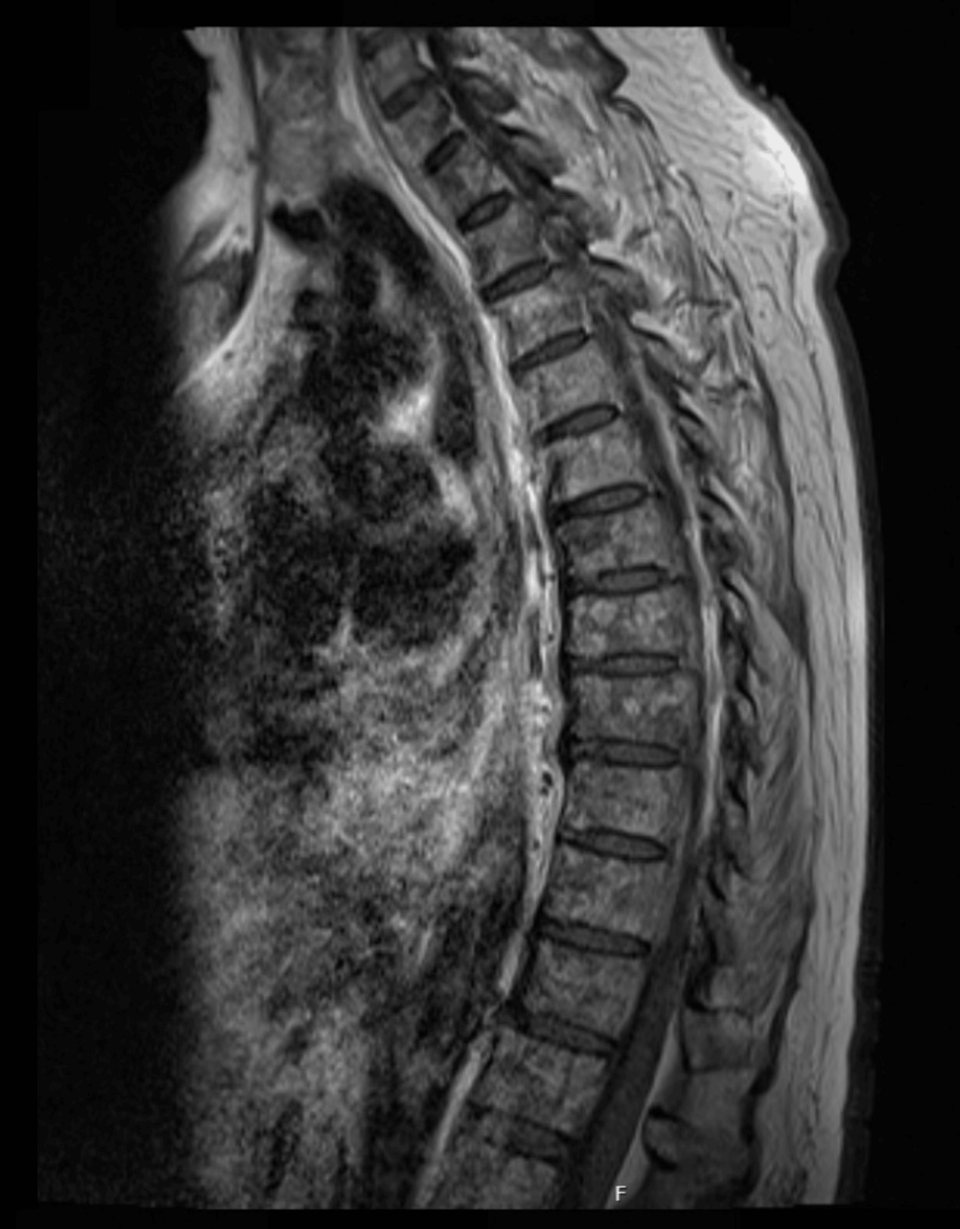
Diffuse alterations in the vertebral bodies and posterior arches of the cervical, thoracic, lumbar, and sacral spine, consistent with spinal metastases

**Figure 2:**
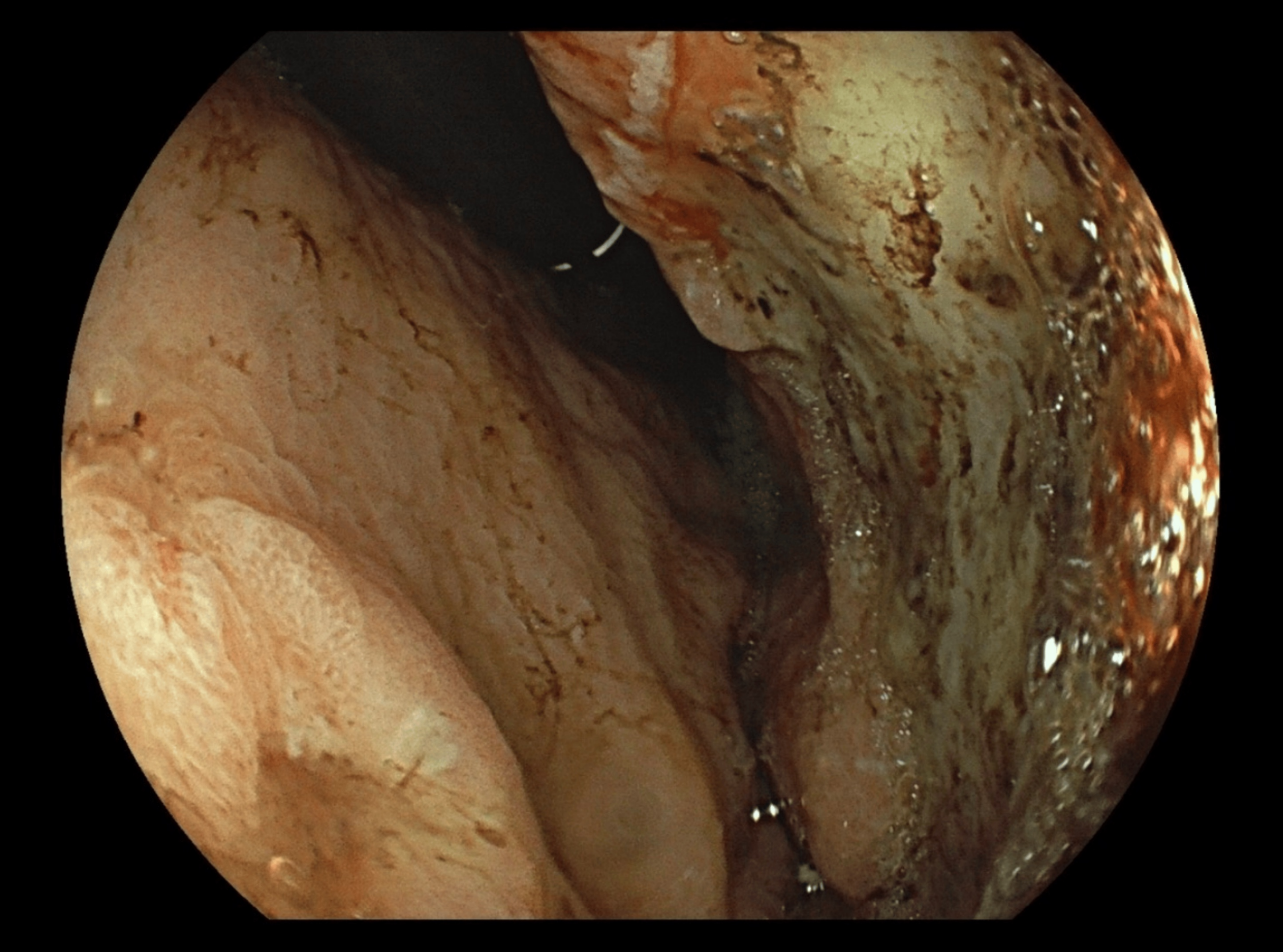
Esophagogastroduodenoscopy demonstrated an ulcerated neoplastic lesion on the anterior wall and greater curvature of the mid-to-distal gastric body

Due to uncontrolled pain and the need for analgesic titration, she was maintained on paracetamol 3 g/day and transdermal buprenorphine 52.5 micrograms. Given the ongoing severe lumbar and cervical pain and the microbiological findings, an MRI was undertaken to investigate a possible spinal focus. The MRI excluded spondylodiscitis but revealed diffuse changes in the vertebral bodies and posterior elements of the cervical, thoracic, lumbar, and sacral spine, highly suggestive of metastatic disease (Figure 1). Upper gastrointestinal endoscopy revealed an ulcerated lesion on the anterior aspect and greater curvature of the mid-to-distal gastric body (Figure 2). Colonoscopy was unremarkable. Biopsy confirmed signet ring cell gastric adenocarcinoma. 

The case was discussed with the general surgery and gastroenterology teams. The gastric neoplasm was considered potentially resectable, though metastatic disease remained uncertain. Endoscopic treatment was not feasible, and given the absence of high-output bleeding, coagulation optimization was prioritized, with sucralfate planned once oral intake resumed.

On hospital day seven, following a 16-day course of ceftriaxone and negative follow-up blood cultures, the patient developed sustained hypotension and worsening renal function (creatinine 2.35 mg/dL, normal values 0.67-1.17 mg/dL). Hemoglobin dropped to 5.4 g/dL with thrombocytopenia of 51×10³/µL. CT imaging showed gastric distension with multiple nodular hyperdense areas consistent with clots, without active bleeding. Upper gastrointestinal endoscopy identified a large clot over the previously detected gastric neoplasm, with no evidence of active bleeding. The patient was transferred to the intensive care unit (ICU) for hemorrhagic shock secondary to upper gastrointestinal bleeding from the gastric ulcer. She received four units of packed red blood cells, with no further gastrointestinal bleeding. Pantoprazole infusion was continued for two days, then switched to intermittent bolus dosing with metoclopramide 10 mg twice daily.

Following ICU stabilization, the patient returned to the internal medicine ward. A follow-up spine MRI showed no improvement in bone lesions, making *Salmonella*-related bone metastasis less likely. Bone biopsy confirmed infiltration of the marrow by signet ring cell gastric adenocarcinoma. Brain CT excluded cerebral metastases. The case was reviewed by the multidisciplinary team, and due to progressive clinical decline, she was deemed unsuitable for systemic therapy and was transitioned to palliative care.

The final diagnosis was gastric adenocarcinoma with isolated bone metastasis, initially detected following NTS bacteremia. Despite all the care provided, the patient experienced progressive clinical and functional deterioration. On the 40th day of hospitalization, the patient’s condition deteriorated, culminating in cardiorespiratory arrest, resulting in death.

## Discussion

NTS bacteremia is an uncommon presentation, typically occurring in immunocompromised individuals. While most NTS infections manifest as self-limiting gastroenteritis, bacteremia develops in 3%-8% of cases, particularly in those with HIV, diabetes, hematological disorders, or gastrointestinal malignancies [2-6]. Although gastric cancer commonly metastasizes to the liver, peritoneum, and lymph nodes, bone involvement is rare, occurring in only 1.5%-5% of cases, and typically in the context of disseminated disease [11,12]. It is more frequently observed in diffuse-type histology and may be associated with a poorer prognosis [11,12]. Isolated bone metastasis, as observed in this patient, is exceptionally rare [11,12].

In this report, we present a rare clinical case of gastric cancer with an atypical presentation, characterized by isolated bone metastasis, in which the diagnosis was prompted by an unusual cause of bacteremia involving NTS.

The association between invasive *Salmonella* infection and malignancy is multifactorial. Cancer-related immunosuppression may result from tumor-induced immune dysfunction, compromised mucosal barriers, and chronic inflammation [7]. Furthermore, *Salmonella* species possess a unique ability to colonize malignant tissues, potentially contributing to disease progression [8].

This report highlights the importance of heightened clinical suspicion in patients with unexplained NTS bacteremia, particularly those without obvious immunosuppression or gastrointestinal symptoms. While NTS infections are often self-limiting, primary bacteremia should prompt an evaluation for an underlying malignancy. A comprehensive diagnostic approach, including imaging and tissue biopsy, is essential for early detection and appropriate management, potentially improving patient outcomes. The patient’s clinical course also underscores the importance of multidisciplinary management.

## Conclusions

This case highlights NTS bacteremia as an important clue to an underlying malignancy or other immunosuppressive risk factor, particularly in patients without obvious immunosuppression. Clinicians should maintain a high index of suspicion and conduct thorough investigations, including imaging and biopsy, when faced with unexplained *Salmonella* bacteremia. Early detection of associated malignancies, such as gastric cancer with isolated bone metastases, is crucial to guide appropriate management and improve patient outcomes. Multidisciplinary collaboration remains essential to optimize care in these complex cases.
